# Local Structure of Ca^2+^ Alginate Hydrogels Gelled via Competitive Ligand Exchange and Measured by Small Angle X-Ray Scattering

**DOI:** 10.3390/gels5010003

**Published:** 2019-01-09

**Authors:** Kyoko Yamamoto, Yoshiaki Yuguchi, Bjørn Torger Stokke, Pawel Sikorski, David C. Bassett

**Affiliations:** 1 Graduate School of Engineering, Osaka Electro-Communication University, 18-8 Hatsu-cho, Neyagawa, Osaka 572-8530, Japan; de17a001@oecu.jp (K.Y.); yuguchi@osakac.ac.jp (Y.Y.); 2 Dept of Physics, NTNU, The Norwegian University of Science and Technology, 7491 Trondheim, Norway; bjorn.stokke@ntnu.no (B.T.S.); pawel.sikorski@ntnu.no (P.S.); 3 School of Chemical Engineering, University of Birmingham, Birmingham B15 2TT, UK

**Keywords:** alginate, ionotropic gelation, competitive ligand exchange crosslinking, small angle X-ray scattering

## Abstract

Alginates, being linear anionic co-polymers of 1,4-linked residues β-d-ManA (M) and α-l-GulA (G), are widely applied as hydrogel biomaterials due to their favourable in vivo biocompatibility and convenient ionic crosslinking. The “egg-box” model is the prevailing description of the local structure of junction zones that form between the alginate chains and divalent cations, such as Ca^2+^, when ionic gelation occurs. In the present study we address to what extent signatures of lateral dimerization and further lateral association of junction zones also represent a valid model for the gelation of alginate using the recently reported method of competitive ligand exchange of chelated Ca^2+^ ions as a method for introducing gelling ions at constant pH. Small angle X-ray scattering with a *q* range from 0.1 to 3.3 nm^−1^ was employed to determine local structure in the hydrogel, using a custom-made fluid sample cell inserted in the X-ray beam. The scattering volume was intended to be localized to the contact zone between the two injected aqueous alginate solutions, and data was captured to resolve the kinetics of the structure formation at three different conditions of pH. The data show evolution of the local structure for the Ca^2+^ induced formation of junction zones in an alginate with 68% G residues, characterized by cross-sectional radii that could be accounted for by a two-component, broken rod like model. The evolution of the two component weight fractions apparently underpinned the connectivity, as reflected in the rheological data.

## 1. Introduction

Alginate is a hugely important biopolymer that has found diverse applications in many products including various foods [1] (mainly as a thickening agent), cosmetics [2], pharmaceuticals [3], wound dressings [4], dental impression materials [5], and textiles [6] among others. An exciting and active contemporary research domain for alginate is within regenerative medicine and tissue engineering as a mimic for the extracellular matrix [7]. This biopolymer is particularly suited for cell encapsulation since it has proven in vitro cytocompatibility [8] and favourable properties regarding human biocompatibility [9,10]. In this regard it has been extensively researched for the encapsulation of islets of Langerhans for the treatment of diabetes [11,12] and for a wide variety of cell, cell spheroid and organoid encapsulation, including device assisted tissue model applications [13,14,15,16,17].

Alginates are an anionic structural polysaccharide extracted from either bacteria or seaweed sources, which vary in overall molecular weight and relative ratios of the (1-4)-linked β-d-mannuronate (M) and its C-5 epimer α-l-guluronate (G) residues that make up the copolymer structure. Aqueous solutions of alginate may be conveniently crosslinked via the formation of junction zones between homopolymeric regions of negatively charged G residues (G-blocks) by multivalent cations to form rigid hydrogels, typically with between 0.5–5 wt/vol% polymer. The strength of alginate gels formed by various multivalent cations depends both on the type of ion and the particular ionic binding property of the alginate, which in turn depends upon its M-G content and monomer sequence. In this regard, it has been reported that guluronic sequences of alginate display binding strengths towards some divalent cations in the order Ba^2+^ > Sr^2+^ > Ca^2+^ >> Mg^2+^, whereas for M-blocks the order is Ba^2+^ > Sr^2+^ ~ Ca^2+^ ~ Mg^2+^, and for alternating GM sequences no significant differences between these ions were observed [18]. By far the most widely applied gelling cation is Ca^2+^, since this ion provides a relatively strong bridging interaction between polymer chains and is not toxic. Ba^2+^ is also commonly added in combination with Ca^2+^ to enhance stability of the hydrogel network, however it is applied very sparingly due to the potent toxicity of this ion [19].

**Figure 1:**
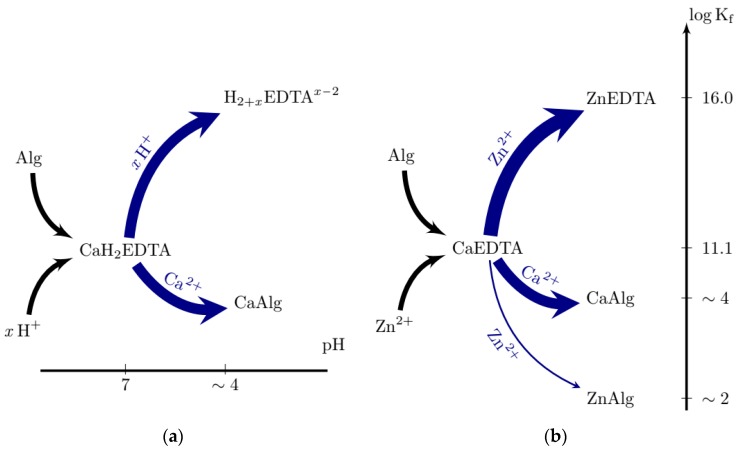
Schematic illustration of exchange reactions employed for ionic gelation of alginate when using the combination of CaEDTA and an acidifying agent, typically glucono δ-lactone (GDL) (**a**), and the competitive ligand exchange reaction, CLEX for the CaEDTA–Zn(EDDA) gelation ion chelator and exchange ion chelator pair (**b**).

Hydrogels formed by the addition of an exogenous aqueous ion such as Ca^2+^ are crosslinked almost instantaneously and as a result, highly inhomogeneous gels are formed. We recently studied the flux of Ca^2+^ and alginate during such gelation in detail using confocal microscopy, interested readers are directed here to ref. [20]. In order to form homogeneous gels, particularly on a large (i.e., >mm) scale, several “internal” gelation systems have been developed. These systems rely on an intrinsic or externally applied trigger to release an internal ionic source that is either chelated, caged or present as a dissolvable solid. Examples of such triggers include pH, light (typically Ultra-Violet) and dissolution; common sources of internal Ca^2+^ include solid calcium minerals such as CaSO_4_(s) [21] or CaCO_3_(s) [22], chelate complexes such as Ca-ethylenediaminetetra-acetic acid (CaEDTA) [23] or Ca-ethyleneglycol tetra-acetic acid (CaEGTA) [24] and “caged calcium” held in photo-labile organic molecules [25]. Of these examples perhaps the most widely used relies on the hydrolysis of glucono δ-lactone (GDL) to provide a source of H^+^ to either dissolve CaCO_3_(s) or substitute Ca^2+^ in Ca-EDTA/EGTA complexes when pH < ~ 5 (Figure 1a).

While these methods are often convenient for certain applications, they rely on the spontaneous hydrolysis of GDL which, therefore, must be prepared fresh and applied immediately. In addition, a significant pH reduction must occur in the case of chelated Ca^2+^. The Ca^2+^ is released from the chelator upon decreasing pH due to the reduced association constant towards the chelator. When applied for the ionic gelation of alginate, one should reduce the pH to about 4 to reduce the fraction of the EGTA/EDTA associated Ca^2+^ while at the same time allowing dominance of the ionic gelation (Ca-Alg) and not the acidic gelation mechanism [26]. When CaCO_3(s)_ is used as the Ca^2+^ source the pH is buffered to near neutral; however, the reaction is accompanied by the evolution of CO_2(g)_ and H_2_O_(l)_ which may significantly alter the properties of the resulting hydrogel and for certain applications, e.g., microfluidics or injectable solutions, the presence of a solid component is not practical. Photolabile molecules are typically very expensive and the use of UV may damage or alter sensitive biological molecules including DNA and RNA.

To overcome the drawbacks associated with conventional internal gelation systems, we were recently motivated to develop a new approach to form hydrogels of ionotropic polymers, such as alginate, that enables precise control over the kinetics crosslinking via a concept we term Competitive Ligand EXchange crosslinking (CLEX) [27]. CLEX has significant advantages over other approaches and is more flexible, particularly since the pH may be set and remains stable over the course of the reaction and all of the reactants remain in the aqueous phase throughout. Briefly, CLEX relies on relative differences in affinity between alginate and soluble chelator species to two different multivalent cations (Figure 1b). The system relies on the mixing of two alginate solutions, one that contains the crosslinking ion (CI) chelated to a species that has a higher affinity to CI than alginate and another that contains an exchange ion (EI) chelated to another species that has a higher affinity to the EI, but lower to the CI than alginate. In the illustrated example (Figure 1b) the affinity of Zn^2+^ towards the EI chelator (EIC), is less than towards alginate and the CI chelator (CIC), e.g., EDTA. These states are illustrated by placing them on the vertical scale corresponding to their association constant (Figure 1b). When the two solutions are mixed, the EI exchanges with the CI, which is rendered free to crosslink the alginate. The kinetics of the reaction can be modified by varying the chemical species involved and the pH of the reaction. We have found particular utility of this approach for the on-chip gelation of microbeads of alginate via droplet based microfluidics and demonstrated excellent viability of both eukaryotic and prokaryotic cells encapsulated using this approach [15].

The first model applied to describe the gelling mechanism of alginate was the “egg-box” model, so called because the dimerised alginate G-blocks form a mirrored zig-zag pattern around a central ion, thus resembling eggs in an eggbox [28]. However, this model only truly describes the initial association and conditions of low fractional Ca^2+^ saturation of low G content alginates. Previously we applied small angle X-ray scattering (SAXS) to determine further lateral association in conditions of higher Ca^2+^ saturation and G content [24,29]. In these previously reported SAXS studies, gelation was facilitated via the widely employed pH dependent release of Ca^2+^ from CaEGTA chelate complexes or CaCO_3_. To date, no ultrastructural characterisation of ionotropic alginate gels employing the CLEX procedure has been reported. In the present work, we report on novel ultrastructural determinations of ionotropic alginate hydrogels employing the CLEX method with an emphasis on the polymer chain association during CLEX gelation as well as apparent equilibria achieved using different conditions of pH.

## 2. Results and Discussion

**Figure 2:**
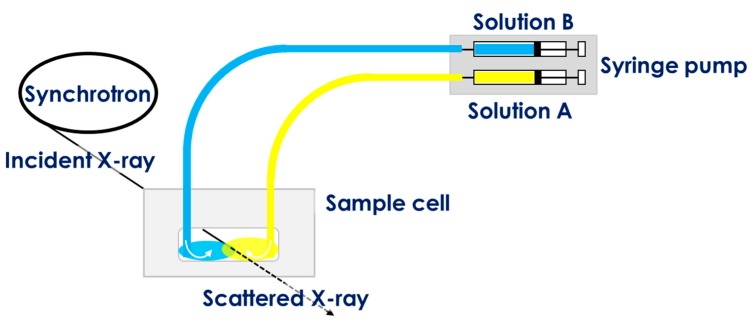
Schematic illustration of the custom sampling system designed to enable time-resolved small angle X-ray scattering (SAXS) measurements of alginate samples.

CLEX gelation is initiated upon contact of EIC and CIC containing alginate solutions. In order to capture the initial gelation events using time-resolved SAXS, a custom sampling system that enabled remote injection of these two solutions was designed (Figure 2). All components of the apparatus resided within the experimental hatch. The two CLEX solutions were injected into the sample cell using syringe pumps allowing for an interface between them to be created at the centre of the mica cell window. The incident X-ray irradiated the centre of the cell perpendicular to the axis of the inlets. This set-up allowed continuous monitoring of time-resolved SAXS intensities at five second intervals before and after injection of the solutions. After a series of measurements, the contact point of the two CLEX solutions was determined by checking the SAXS profiles.

**Figure 3:**
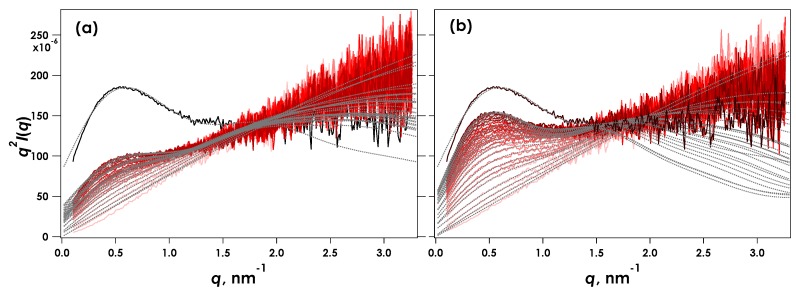
Kratky plots obtained for time-resolved SAXS during CLEX gelation of 1% alginate in aqueous MOPS buffer including CaEDTA and ZnEDDA at Ca^2+^ = 30 mM and pH = 7.0. The results (**a**) and (**b**) correspond to independent measurements under the same experimental conditions. The profiles show data recorded every 5 s and the total trace times are 145 and 130 s for (**a**) and (**b**) respectively. Solid black lines indicate SAXS obtained from fully reacted samples following 10 h reaction time.

Figure 3 shows the Kratky plots (*q^2^I*(*q*) vs. *q*) obtained for the time-resolved SAXS measurements during CLEX gelation of 1% alginate in aqueous MOPS buffer containing CaEDTA and ZnEDDA at Ca = 30 mM and pH = 7.0. Here *I*(*q*) is the scattering intensity and *q* is the magnitude of the scattering vector defined by (4π/λ)sinθ, where λ is the wavelength of the incident X-ray and 2θ is the scattering angle. The time-resolved measurement under the same condition was carried out twice as shown in Figure 3a,b. Initially, immediately following injection of the sample, the Kratky plots showed a linear increase in *q^2^I*(*q*) with *q*, suggesting that the alginate chain was in a single coil condition. 

A maximum gradually appeared around *q* = 0.5 nm^−1^ indicating association of alginate chains. This feature is most clearly discernible in the data within the first two minutes of the experiment. This behaviour indicates that our custom sampling system was effective in enabling measurements of gelation events that occur on a time scale of seconds. The scattering observed following extended equilibration of the sample (depicted “Full gel”, Figure 3) showed a more clearly developed peak around *q* = 0.5 nm^−1^. The apparent difference in the data collected from two independent, parallel experiments (Figure 3a,b) was thought to originate from different solution contact points with respect to the incident X-ray beam. Note also that no agitation or mixing of the sample within the cell following injection were made, therefore the reaction was primarily driven via diffusion of reactants from the contact point of the two solutions. Despite this potential drawback, these results can be used to elucidate the gelation dynamics. The SAXS profiles were analysed by a broken rod model composed of two cylinders with different radii as shown in Equation (1) below.

(1) $$q^{2}I\left(q\right)\approx qk_{1}{\left[\frac{J_{1}\left(qR_{c1}\right)}{qR_{c1}}\right]}^{2}+qk_{2}{\left[\frac{J_{1}\left(qR_{c2}\right)}{qR_{c2}}\right]}^{2}+const.$$

where the *R*_c__i_ is the cross-sectional radius of *i*-th component, *J*_1_(*x*) is the first order Bessel function, and *k*_i_ is a constant relating the weight fraction of the rod component. The fitted curves for the data shown in Figure 3 indicate that the broken rod model with two components (Equation (1)) adequately accounted for the experimental observations. The deviation in *q* > 2 nm^−1^ observed at more extended reaction times could arise from the emergence of a broader distribution of lateral association modes in the junction zones than those that are well accounted for by the two-component model.

**Figure 4:**
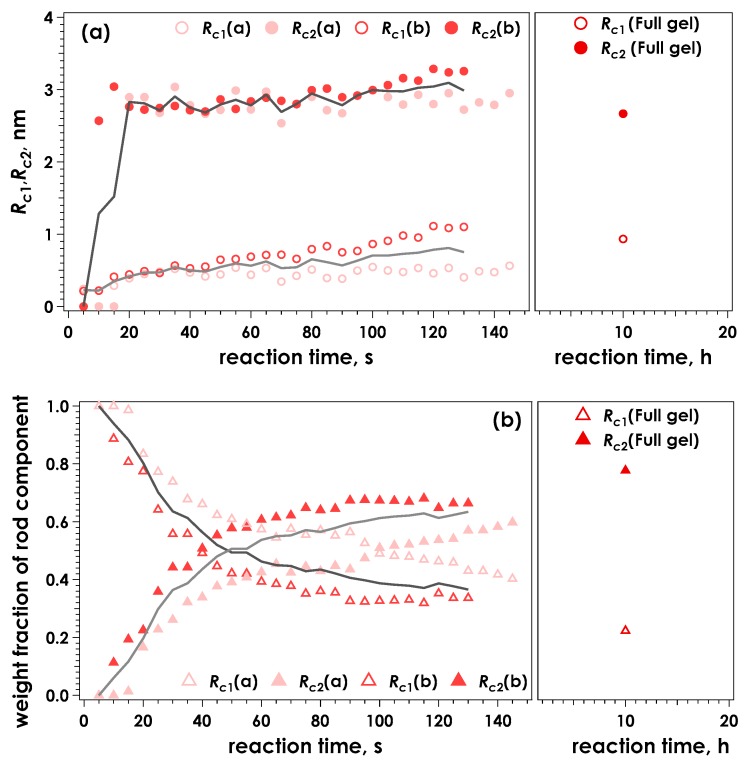
Time course for the calculated radii of the two rod components, *R*_c1_ and *R*_c2_ (**a**) and the corresponding weight fractions of the two components (**b**) determined by evaluation of the data presented in Figure 3 using a broken rod model. The solid lines indicate the averaged values for the two measurements.

Figure 4 shows the radii of the rod components and their weight fractions as a function of time for the data shown in Figure 3, calculated using the two-component broken rod model. One rod component was determined to have a radius of 0.2 nm in the initial stage of reaction, suggesting the alginate molecules are initially dispersed as single chains. 

Following this initial value, an increase in cross-section of the thinnest component (*R*_c1_) was observed, resulting in an average value of approximately 0.9 nm for the equilibrated gel. A second, thicker component (*R*_c2_) was clearly discernible from 10 s following sample injection onwards. The radius of this component was around 2.6–3.0 nm, and was observed to increase less throughout the experiment, compared to *R*_c1_. Similar to our previous analysis of SAXS data obtained for the ionotropic gelation of alginate [24,29], it is suggested that this component is mainly due to the junction zones being formed. Changes in weight fractions occur rapidly within the first two minutes, followed by a slower, less substantial change observed after 10 h reaction time. The larger components constitute 60% after two minutes, representing 60% junction zones and 40% chains connected with them. This fraction increased to approximately 80% after 10 hours. An almost identical result was obtained for samples gelled using a slowly hydrolysing lactone (GDL) to gradually lower the pH of an alginate solution containing 30 mM CaEDTA and release the chelated Ca^2+^ (denoted CaEDTA-GDL, see supplementary information and Figures S1 and S2). Therefore at this condition of pH, complete exchange of ions had occurred and the alginate was likely completely saturated with Ca^2+^. 

**Figure 5:**
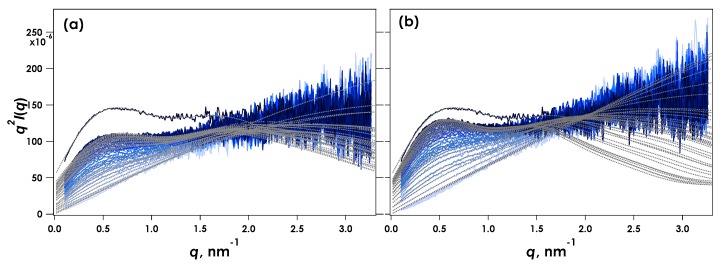
Kratky plots obtained for time-resolved SAXS during CLEX gelation of 1% alginate in aqueous MOPS buffer including CaEDTA and ZnEDDA at Ca^2+^ = 30 mM and pH = 7.4. The results (**a**) and (**b**) correspond to independent measurements under the same experimental conditions. The profiles show data recorded every 5 s and the total trace times are 175 and 180 s for (**a**) and (**b**) respectively. Solid black lines indicate SAXS obtained from fully reacted gel samples following 10 h reaction time.

**Figure 6:**
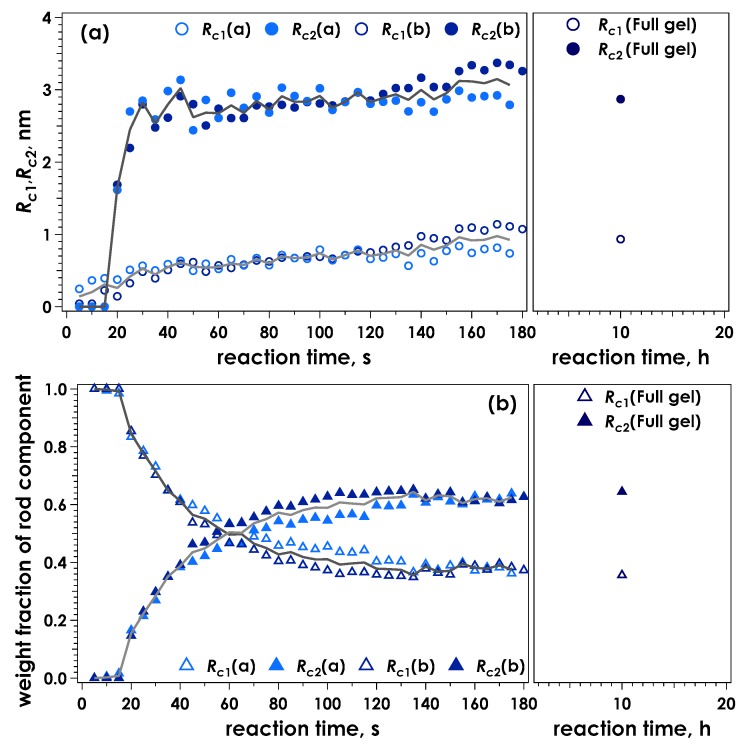
Time course for the calculated radii of the two rod components, *R*_c1_ and *R*_c2_ (**a**) and the corresponding weight fractions of the two components (**b**) determined by evaluation of the data presented in Figure 5 using a broken rod model. The solid lines indicate the averaged values for the two measurements.

Figure 5 shows the Kratky plots for SAXS data obtained from CLEX gelled alginates at pH = 7.4. Though the SAXS data obtained was similar to that for pH = 7.0 (c.f. Figure 3), the maximum *q^2^I*(*q*) obtained from the fully reacted gelled sample at 10 h reaction time was smaller than at pH = 7.0, suggesting that the alginate chains were not fully associated and crosslinking was not complete. Figure 6 shows the calculated radii of the rod components and their weight fractions obtained using the two-component broken rod model as a function of time for the data shown in Figure 5. Again, the results were very similar to the pH 7.0 condition (c.f. Figure 4), with *R*_c1_ and *R*_c2_ values of 0.93 and 2.9 nm respectively. A slightly longer time was required to achieve a weight fraction greater than 60% of the larger R component (*R*_c2_), and unlike pH 7.0 this fraction remained similar following 10 h reaction time and did not substantially increase. This behaviour indicates that the exchange reaction was not complete and a proportion of the Ca^2+^ remained chelated with EDTA or was associated with the EIC, EDDA.

**Figure 7:**
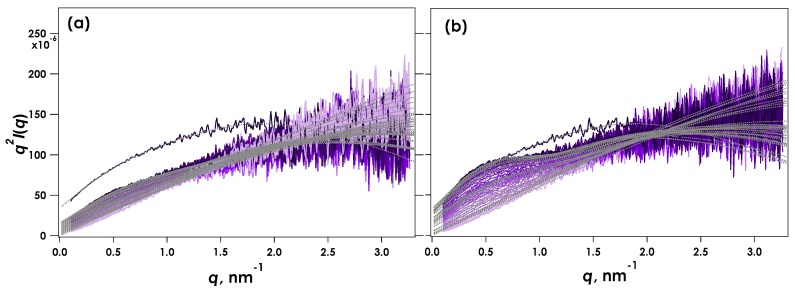
Kratky plots obtained for time-resolved SAXS during CLEX gelation of 1% alginate in aqueous HEPES buffer including CaEDTA and ZnEDDA at Ca^2+^ = 30 mM and pH = 8.0. The results (**a**) and (**b**) correspond to independent measurements under the same experimental conditions. The profiles show data recorded every 5 s and the total trace times are 180 s. Solid black lines indicate SAXS obtained from fully reacted samples following 10 h reaction time.

**Figure 8:**
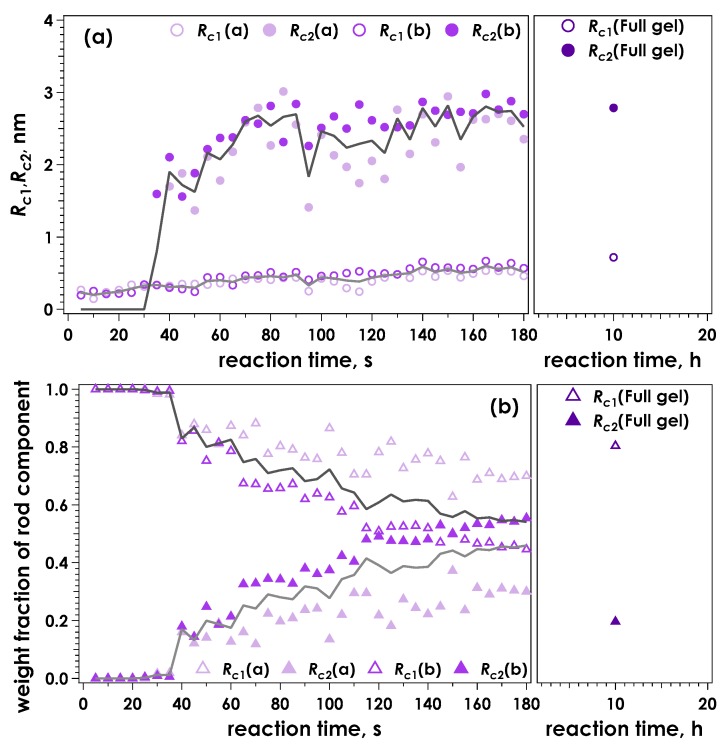
Time course for the calculated radii of the two rod components, *R*_c1_ and *R*_c2_ (**a**) and the corresponding weight fractions of the two components (**b**) determined by evaluation of the data presented in Figure 7 using a broken rod model. The solid lines indicate the averaged values for the two measurements.

A further increase in pH resulted in much weaker SAXS profiles and no clear maximum was discernible following 10 h reaction time. Figure 7 shows the SAXS data obtained from CLEX alginate samples gelled at pH = 8.0 and indicates that the alginate chains aggregated very weakly and gelation was not complete. However, as shown in Figure 8 some junction zones did form as evidenced by the clear appearance of an *R*_c2_ value of 2.5 nm, comparable to the other conditions of pH. However, the reaction was much slower, requiring 180 s to achieve almost 50 wt% of the *R*_c1_ component. After 10 h reaction time this fraction had reduced to approximately 25 wt%. This behaviour may seem curious but is explained by considering the relative amount of Ca^2+^ available to interact with the alginate within the scattering zone, which is initially high, and then reduces as the two solutions diffuse into each other. Since in this condition of pH a large proportion of the Ca^2+^ remains chelated by EDTA or is exchanged with Zn^2+^ and becomes chelated with EDDA, there is less available to form junction zones with the alginate compared to the conditions of lower pH. By 10 h, diffusion of the reaction components has likely been completed. At this stage and under these conditions, the apparent fraction of the larger *R*_c2_ component, representing the formed junction zones has halved the compared values recorded shortly after contact of the two CLEX components. Since at pH = 8.0, a majority of the Ca^2+^ is chelated and not available for gel formation, this decrease in the fraction of the *R*_c2_ component could be caused by slow gel reorganisation, for example via the formation of fewer but more Ca-rich junction zones.

**Figure 9:**
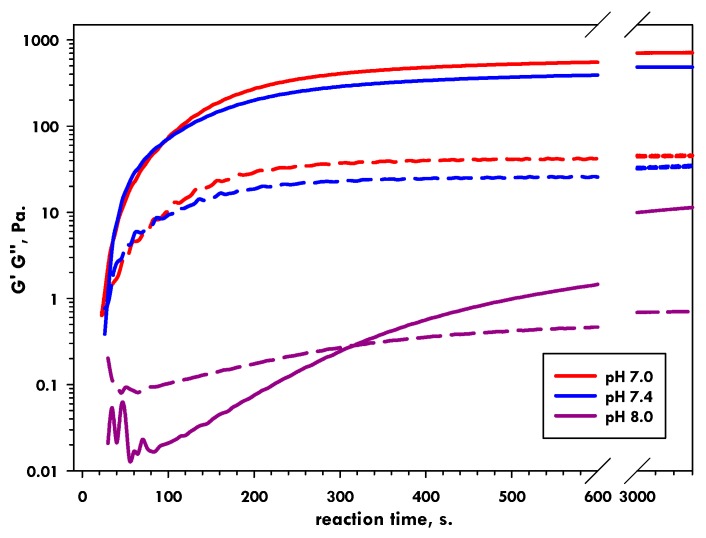
Rheological measurements of 1% CLEX alginates gels at the indicated pH. Solid lines = G’, dashed lines = G’’. (Data originally reported in [27]).

**Figure 10:**
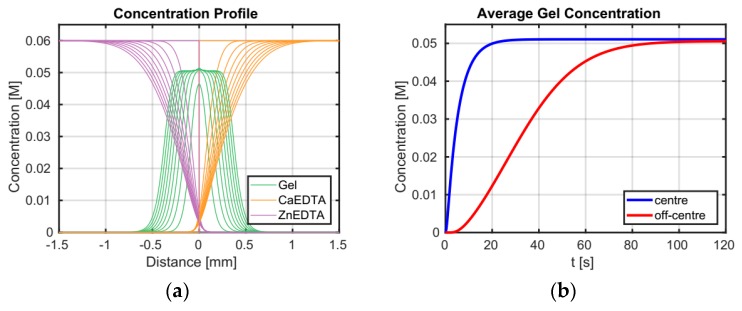
(**a**) Calculated concentration profiles for CLEX components and alginate gel at the contact zone plotted every 12 s for the first 120 s of the gel formation process. (**b**) Evolution of gel concentration at the junction of the two CLEX solutions (region of interest is 50 µm across) and at an off-centre position (offset of 200 μm) to illustrate the difference in the gel formation kinetics due to diffusion of the cross-linking ions at these two locations. The gel concentration is expressed in terms of moles of monomer per litre. Observed concentration profiles are in qualitative agreement with SAXS data obtained at pH 7, where the used model correctly describes the exchange reaction whereby the exchange reaction in complete. At higher pH not all calcium supplied as CaEDTA is released and made available for gel formation.

The SAXS analyses perfectly complement our previous rheological observations regarding the effect of increasing pH on the CLEX gelation of alginate (Figure 9) [27]. Here we observed the same trend of decreasing gel strengths and increasing gelation times as a function of increasing pH. As briefly explained above, we believe this occurs as an equilibrium in the affinity for the CI (i.e., Ca^2+^) between the EIC (i.e., EDDA) and alginate is approached as the pH is increased. The SAXS results provide clear evidence that the formation of junction zones is perturbed as a function of pH and this is almost certainly due to a restriction in the availability of Ca^2+^ to alginate. The time scales of reaction observed using SAXS and rheology differ slightly, but the trends remain the same. Here the kinetics of the reaction are primary controlled by the diffusion of CLEX components into the observation volume which takes place on the minute timescale. The exchange reaction itself occurs on the timescale of seconds, as shown by gel formation kinetics in our rheology measurements where the contact zone between the two CLEX solutions was proportionally much larger than in the custom SAXS chamber used in these experiments [27]. Differences in diffusional behaviour also likely explain the initial differences observed between individual SAXS measurements under the same conditions, since after a prolonged reaction time of 10 h, little difference in scattering behaviour was found between samples. Ca^2+^ is released close to the contact zone, where the two CLEX solutions meet. The released Ca^2+^ ions then gel the alginate as they diffuse outwards increasing the volume of formed gel. Therefore the differences in repeat SAXS measurements were likely enhanced the further away the contact point between the two CLEX solutions was from the incident beam path; we modelled this effect and show the results in Figure 10 to illustrate this concept.

## 3. Conclusions

For the first time we dynamically examined the local structure of alginate gelled using a newly described gelation mechanism that relies upon on competitive ligand exchange of chelated Ca^2+^ ions. Small angle-X-ray scattering provided clear evidence for the evolution of junction zones that could be well accounted for using a two-component broken rod model. The resulting proportion of the two component weight fractions reflected the degree of connectivity that was achieved as a function of pH; behaviour that was further supported by rheological data. Otherwise, the local structure appeared unaffected by the chemical components of CLEX, since the size and fraction of fully reacted gels was not substantially different to control gels prepared by the CaEDTA-GDL method.

## 4. Materials and Methods 

All chemicals were purchased from Sigma Aldrich (Oslo, Norway), unless otherwise stated. Pure water (de-ionized or ultra-pure water with a resistivity of 10–18 MΩ cm) was used to prepare all aqueous solutions. Aqueous CLEX alginate solutions were prepared as described previously [27]. CLEX solutions were prepared at pH 7.0, 7.4 and 8.0. Alginate (source L. hyperborea stipe) with a guluronic acid residue fraction of FG = 0.68, corresponding to 68% (PROTONAL LF 2005, FMC Biopolymer, Haugesund, Norway) and a MW of 275 kg mol^−1^ was dissolved in pure water to a final concentration of 3 wt%. In all samples the crosslinking ion chelator (CIC) solutions were prepared by mixing 1 M CaCl_2_ with 0.5 M ethylenediaminetetra-acetic acid (EDTA) (Invitrogen, Oslo, Norway) and 1 M of either 3-morpholinopropane-1-sulfonic acid (MOPS) for pH 7.0 and 7.4 or 4-(2-hydroxyethyl)-1-piperazineethanesulfonic acid (HEPES) for pH 8.0. Samples were then adjusted to the desired pH using NaOH and the concentrations adjusted with pure water to give a final concentration of 60 mM Ca-CIC, 60 mM buffer and 1% hydrogel. The procedure was similar to prepare the Exchange ion chelator (EIC) solutions at pH 7.0, 7.4 and 8.0 by substituting Zn(CH_3_CO_2_)_2_ for CaCl_2_ and ethylenediamine-*N*,*N*’-diacetic acid (EDDA) (Alfa Aesar, Heysham, United Kingdom or Sigma Aldrich, Tokyo, Japan) for EDTA. The resulting concentrations in the final EIC solution were the same as the CIC solution: 60 mM Zn-EIC, 60 mM buffer and 1% hydrogel. Alginate gels were also formed following the method of Draget et al. [22] Briefly, a solution containing 1.5 wt/vol% alginate and 45 mM Ca-EDTA at pH 7.0 was mixed in a 2:1 ratio with a freshly prepared 180 mM aqueous solution of d-glucono-δ-lactone (GDL, Wako Pure Chemical Industries, Ltd., Osaka, Japan).

Small-angle X-ray scattering (SAXS) measurements were performed at the SPring-8 facility (BL-40B2) in the Hyōgo Prefecture, Japan. The wavelength of the incident X-ray beam was 0.1 nm. The scattered X-rays were detected by PILATUS (DECTRIS, Baden, Switzerland) placed about 1 m from the sample holder, yielding magnitudes of the scattering vector (*q*) in the range of 0.103–3.26 nm^−1^. The CLEX solutions were injected into a stainless steel sample cell with entrance and exit windows made of mica by means of a syringe pump (YMC Co., Ltd., Kyoto, Japan). The two CLEX solutions were injected separately at the same rate from opposite ends of the sample holder to enable contact at the centre of the cell window. Single solutions of Ca-EDTA/GDL control gels were injected into one port and allowed to cure inside the sample cell. Buffer solutions containing no alginate were also injected into the sample cell through one port. Time-resolved measurements were performed by repeating a program of 5 s interval including an exposure time of 2.5 s. The scattering profiles were calculated by subtraction of the scattering recorded from buffer solutions from that of sample solutions or gels.

Rheological characterization was performed as described previously [27], and repeated here for clarity. All rheological measurements were conducted using a Paar Physica MCR 300 Rheometer (Graz, Austria). A parallel plate geometry with serrated plate surfaces (PP50 serrated plate, diameter = 50 mm) which provided minimal wall slip was used. Storage and loss moduli at a measuring gap of 1 mm were recorded as a function of time at an angular frequency (*ω*) of 1 rad s^−1^, amplitude of 1 mrad and temperature of 25 °C. Equal volumes (1.75 mL) of 2 component gels were measured onto the rheometer plate using a 5 mL pipette. The first component was placed directly onto the bottom plate, then the other was pipetted onto it just prior to starting the measurement. Low viscosity silicone oil (200/10 cS fluid, Dow Corning, Midland, MI, USA) was introduced following setting of the gap to seal the gel sample from the atmosphere and to prevent evaporation. Using this approach, a lag time of approximately 30 s resulted from the time of delivery of the second polymer solution to the start of data collection. For all measurements of CLEX hydrogels, the final concentration of Ca^2+^, Zn^2+^ and chelators was 30 mM. pH was maintained using pH buffers, pH 7 and 7.4 = MOPS, pH 8 = HEPES. All measurements were repeated a minimum of three times.

The concentrations of the alginate gel and of the CLEX components at the contact zone of the two CLEX solutions were calculated using a previously described alginate gelling model [20]. This model was modified to account for the supply of gelling ion through the CLEX mechanism. Evolution of the concentration profiles of CaEDTA and ZnEDDA was described by a reaction diffusion model in 1D (see Equations (2)–(4)) and the exchange reaction is given by Equation (5).

(2) $$\frac{\partial C_{1}}{\partial t}=D_{1}\frac{\partial^{2}C_{2}}{\partial x^{2}}-\kappa C_{1}\cdot C_{2}$$

(3) $$\frac{\partial C_{2}}{\partial t}=D_{2}\frac{\partial^{2}C_{2}}{\partial x^{2}}-\kappa C_{1}\cdot C_{2}$$

(4) $$\frac{\partial C_{3}}{\partial t}=\kappa C_{1}\cdot C_{2}$$

ZnEDDA + CaEDTA ↔ ZnEDTA + Ca^2+^ + EDDA^2−^ (5)

The *x*-coordinate is perpendicular to the mixing zone direction, *D*_1_ and *D*_2_ are the diffusion constants of CaEDTA and ZnEDDA respectively and *C*_1_ and *C*_2_ are time and position dependent concentrations of CaEDTA and ZnEDDA respectively. Term −*κC*_1_ *C*_2_ describes the reduction in the concentration of CaEDTA and ZnEDDA due to the exchange reaction. This ion exchange process is described by a second order chemical reaction with the rate constant *κ*. Calcium ion concentration available for gel formation (*C*_3_) is calculated using Equation (4). Here the rate constant, *κ,* was set to a value large enough such that the exchange reaction was not a rate limiting step (in the simulation shown in Figure 10, *κ* = 10^4^ M^−1^ s^−1^). Generated calcium ions are able to gel the alginate gel or diffuse in the solution, depending on the local concentration of the ungelled alginate. The calculations were performed using pdepe solver in MATLAB Version: 9.2.0.538062. The simulation box size was chosen such that the concentrations of all components at the boundary were constant and the concentration gradient was zero. The same diffusion constant of 0.78 10^−9^ m^2^ s^−1^ was assumed for CaEDTA and ZnEDDA. All other model parameters were based on those given previously [20]). The Matlab code is available upon request. 

## Supplementary Materials

The following are available online at http://www.mdpi.com/2310-2861/5/1/3/s1.
