# Supplementary material for: Local Structure of Ca2+ Alginate Hydrogels Gelled via Competitive Ligand Exchange and Measured by Small Angle X-Ray Scattering
Source: Gels. 2019 Jan 9;5(1):3. doi: 10.3390/gels5010003 (PMC6473945; doi:10.3390/gels5010003)
Supplement: Supplementary file 1 [file gels-05-00003-s001.pdf]

Supplementary information:

Local structure of Ca^{2+} alginate hydrogels gelled via competitive ligand exchange and measured by small angle X-ray scattering.

Kyoko Yamamoto, Yoshiaki Yuguchi, Bjørn Torger Stokke, Pawel Sikorski and David C. Bassett

Fig S1 shows the time-resolved SAXS result from a slow gelation system driven by the hydrolysis of GDL to acidify a solution of 30mM CaEDTA and 1% alginate. Here the SAXS profile was traced for 1 hour with 10 s exposure and 5 min interval. From rheological measurements, gelation occurs in this system after approximately one hour from mixing gel components. Changes in local structure were observed after only 30 min, however over the first hour these scattering data did not reveal a clear peak in Kratky plots similar to that observed for the CLEX procedure. However, the SAXS from the equilibrium state 10 h after introducing GDL to start the reduction of the pH had a clear peak similar to that observed for the CLEX system crosslinked at pH 7.0. This suggests that the structure of the final gel achieved with GDL-CaEDTA was similar to cross-linking zone of CLEX system; occurring, albeit, over a much longer timeframe.

The scattering profiles were also analysed by application of a broken-rod model in same way as the CLEX samples. According to the evaluated values shown in Fig S2, during the initial stage of reaction (0 - 30 min), a single rod component present in solution that does not develop sufficiently to induce a connectivity transition required for gel formation. After approximately 30 minutes, a second larger rod component appeared indicating the start of chain association.

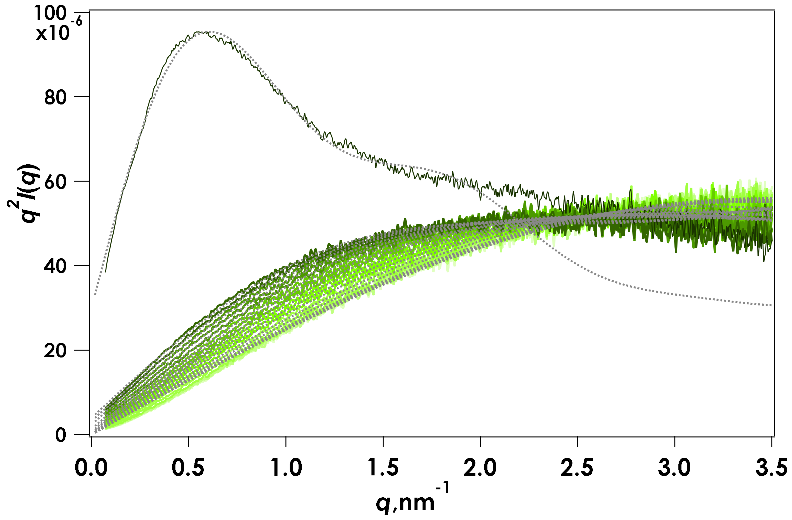

Figure S1. Kratky plots obtained for time-resolved SAXS during slow gelation by addition of GDL to 1% alginate containing Ca-EDTA at Ca^{2+} = 30mM. The profiles show data recorded every 300 s and the total trace time is 3600 s. Solid line indicates SAXS obtained from fully gelled samples following 10 h reaction time.

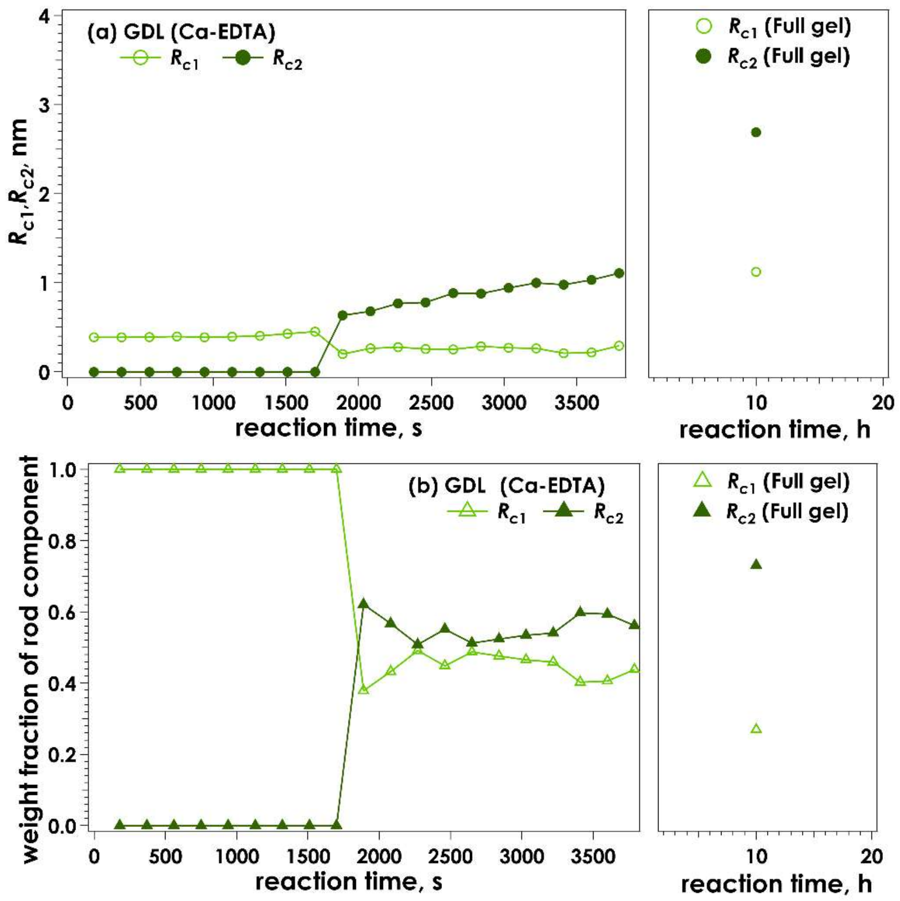

Figure. S2 Time course of the calculated radii of the two rod components, R_{c1} and R_{c2} (a) and the corresponding weight fractions of the two components (b) determined by evaluation of the data presented in figure S1 using a broken rod model.
